# Hospital factors that predict intention of health care workers to leave their job during the COVID‐19 pandemic

**DOI:** 10.1111/jnu.12771

**Published:** 2022-02-20

**Authors:** Yi‐Chuan Chen, Hsueh‐Ching Wu, Feng‐Tze Kuo, David Koh, Yue‐Liang Leon Guo, Judith Shu‐Chu Shiao

**Affiliations:** ^1^ National Institute of Environmental Health Science National Health Research Institutes Miaoli County Taiwan; ^2^ Department of Nursing Hsin Sheng Junior College of Medical Care and Management Taoyuan Taiwan; ^3^ School of Nursing, College of Medicine National Taiwan University (NTU) Taipei Taiwan; ^4^ Pengiran Anak Puteri Rashidah Sa'adatul Bolkiah (PAPRSB) Institute of Health Sciences Universiti Brunei Darussalam Bandar Seri Begawan Brunei Darussalam; ^5^ Saw Swee Hock School of Public Health National University of Singapore Singapore Singapore; ^6^ Department of Environment and Occupational Medicine, College of Medicine National Taiwan University (NTU) and NTU Hospital Taipei Taiwan; ^7^ School of Nursing, College of Medicine National Taiwan University (NTU) and NTU Hospital Taipei Taiwan; ^8^ Susan Wakil School of Nursing and Midwifery The University of Sydney Sydney New South Wales Australia

**Keywords:** COVID‐19, health personnel, health workforce, intention to leave, occupational health

## Abstract

**Purpose:**

To identify factors responsible for hospital health care workers' intention to leave their job during the COVID‐19 pandemic.

**Design:**

A cross‐sectional study was performed.

**Methods:**

A self‐administered questionnaire was delivered to solicit hospital health care workers' demographics, intention to leave, workplace environment, and changes related to COVID‐19 from July to November 2020 in Taiwan. Principal component analysis was performed to compare group‐related factors. Multiple logistic regression was used to determine the risk factors for the intention of health care workers to leave their job.

**Findings:**

Among the 1209 health care workers (mean age, 36.3 years) who participated in the study, intention to leave the job was found to be related to factors relating to COVID‐19, including perceived risk, affected social relationships, and increased workload and job stress, after adjustment for demographic and work factors. Supportive administration/management were protective factors against leaving the job. These results were supported by sensitivity analyses.

**Conclusions:**

Our findings suggest that the intention of health care workers to leave their job during a pandemic is related to potentially modifiable factors relating to the infection itself and work environment.

**Clinical relevance:**

High perceived risk of COVID‐19, affected social relationaops, and increased workload and job stress were positively associated with the intention of health care workers to leave their job, whereas supportive administration and management were protective factors against leaving the job. Development of workplace strategies is important to help mitigate these above factors, improve psychological wellbeing, and promote workforce stability.

## BACKGROUND

The novel coronavirus disease 2019 (COVID‐19), declared by the World Health Organization (WHO) as a pandemic on March 11, 2020, has infected over 170 million individuals and caused more than 3.5 million deaths as at the end of May, 2021 (Dong et al., 2020).

Health care workers are considered a high‐risk group for contraction of the infection owing to their occupations and roles. By April 8, 2020, at least 22,000 health care workers in 52 countries were infected with COVID‐19 (WHO, 2020). In the opening remarks at the World Health Assembly in 2021, 115,000 health care workers have been estimated died from COVID‐19 (WHO, 2021a). During the COVID‐19 pandemic, health care workers are essential human assets worldwide. Therefore, to maintain appropriate staffing during the pandemic is particularly important.

A meta‐analysis focused on health care workers observed that as the pandemic progressed, the percentage of positive COVID‐19 tests among health care workers was 51.7% (95% confidence interval [CI]: 34.7–68.2), and the pooled prevalence of hospitalization and death was 15.1% (95% CI: 5.6–35) and 1.5% (95% CI: 0.5–3.9), respectively (Gholami et al., 2021). In Taiwan, 1072 laboratory‐confirmed cases of COVID‐19 were reported until April 17, 2021. Of the confirmed cases, 77 were indigenous (Taiwan Centers for Disease Control, 2021). A total of 11 patients were infected owing to their work in health care settings. Health care workers have accounted for 14.29% (11/77) of indigenous cases of COVID‐19 in Taiwan.

During the pandemic, health care workers are influenced in many dimensions, including physical health, social well‐being, and working conditions (WHO, 2021b). The risk factors of infection among health care workers included working in high‐risk units, long working hours, and inadequate or inappropriate use of personal protective equipment (Gholami et al., 2021; Ran et al., 2020). In addition to the risk of exposure, they also encounter medical difficulties, increasing routine work procedures, workload, stress, loss of patients and colleagues, fear of infection among their family members, and exhaustion due to work discrimination (Awano et al., 2020). Lai et al. (2020) found that frontline health care workers in China showed symptoms of depression, anxiety, insomnia, and distress with percentages ranging from 40.7% to 76.3%, which were much higher than the corresponding symptoms observed in the second‐line staff (29.3%–68.2%). In addition, it has been reported that during the severe acute respiratory syndrome (SARS) epidemic that occurred a few years ago, 25.9% of nurses looked for another job or considered resigning owing to the risk of SARS and 7.6% of nurses seriously considered leaving their job (Shiao et al., 2007).

As appealed by the Centers for Disease Control and Prevention (2021) in the United States, it is essential to maintain appropriate staffing in health care facilities for health care workers and patients. Therefore, potential staffing shortages and corresponding strategies should be planned. Norkiene et al. (2021) reported that higher levels of stress and lower psychological wellbeing were associated with stronger ideation to leave among health care workers. Thus, it is crucial to identify the risk factors and develop strategies to prevent the resignation of health care workers.

Through this study, we aimed to identify hospital factors that could predict the intention of health care workers to leave their job during the COVID‐19 pandemic.

## METHODS

### Study population

Participants were randomly sampled from 464 hospitals awarded accreditation of conformity or excellence by the Joint Commission of Taiwan between 2013 and 2016. Full‐time hospital health care workers, including doctors, nurses, pharmacists, physical therapists, occupational therapists, respiratory therapists, medical technologists, radiologists, and social workers, were invited to participate in the survey. The sample size was estimated using two‐sided logistic regression test using G*Power, version 3.1.9.7. The conditional probabilities under null hypothesis and alternative hypothesis were calculated according to the relationship between nurses' job tenure and consideration of leaving their job (Shiao et al., 2007), and resulted in 0.096 and 0.055, respectively. The probability of making a type I error was set for 0.05, and the power was 0.9. The proportion of variance explained by additional predictors in the model was assigned as 0.25 for assuming the medium correlation between job tenure and other predictors (Yenipinar et al., 2019). Therefore, the estimated sample size was 682. To account for a 40% loss from invalid questionnaires, the required sample size was 1136.

### Study design

A cross‐sectional study using a self‐administered questionnaire was conducted from July to November 2020 in Taiwan.

#### Measures

#### Workplace environment and risks of COVID‐19

The questionnaire was referred from and authorized to use by Shiao et al. (2007). It consisted of 26 items to investigate the participants' workplace environment and their perception of risk of contracting COVID‐19. All items were scored on a Likert scale (1 = “strongly disagree” to 4 = “strongly agree”). A higher score indicated a stronger perception of risk experienced by a participant.

#### Intention of health care workers to leave

Two statements solicited the commitment of the health care workers to their job during the COVID‐19 progress, namely “I feel that I should not be looking after patients with COVID‐19,” and “I am looking for another job or considering resigning because of the risk.” Participants who agreed/strongly agreed with these two statements were defined as those “seriously considering leaving their job.”

#### Potential confounders

According to previous studies, several potential confounders were considered in this study. Demographics included age, work tenure, and marital status (Norkiene et al., 2021; Shiao et al., 2007). Characteristics related to the practice environment included ownership and level of the hospital, unit, and weekly working hours (Gholami et al., 2021; Ran et al., 2020). The potential confounders were classified as hospital ownership (public, private), hospital level (primary, secondary, tertiary), age (≤25, 26–35, ≥36 years), work tenure (<5, 5–10, >10 years), marital status (single/divorced/widowed, married), and weekly working hours (≤50, >50).

#### Validity and reliability

Expert validity was assessed by three nursing professionals. Items of the questionnaire were scored on a Likert‐type scale (1 and 2: modification required; 3: related; 4: strongly related). After alteration or deletion of inadequate items (scores lower than or equal to 2), the content validity index of the questionnaire was 0.97.

Test–retest reliability were examined by analyzing Pearson's correlation with a 2‐week interval. A convenience sample of 31 hospital health care workers in Taiwan was invited for test–retest reliability; 24 pairs of questionnaires were completed. The test–retest reliability of the questionnaire as a whole was 0.64.

### Statistical analysis

Descriptive statistics were calculated to summarize the demographic characteristics of the participants. Principal component analysis was performed to evaluate and classify the major components of the risk perceptions of the health care workers. The potential confounders were included in the multivariable‐adjusted models to further identify factors important for predicting the intention of health care workers to leave their job. The association between risk factors and outcome was presented as odds ratio and 95% CI. Data were analyzed using two‐sided tests using JMP, version 10.0 (SAS Institute). A *p* value of <0.05 indicated statistical significance. Sensitivity analyses were performed to validate the robustness of the main study findings.

### Ethical consideration

The study protocol was approved by the National Taiwan University Hospital Research Ethics Committee. The requirement of obtaining written consent from the participants was waived, and the returning of completed questionnaires indicated the willingness of the health care workers to participate in the study.

## RESULTS

A total of 1359 questionnaires were disseminated to eight tertiary, 14 secondary, and 21 primary hospitals; 1246 of them were completed satisfactorily and returned. Questionnaires in which more than one‐third part was incomplete were excluded, leaving 1209 eligible questionnaires for the final analysis. The total effective response rate was 88.9%.

The participants' demographics are summarized in Table S1. The majority of the study population was female (*n* = 992, 82.1%), with a mean age of 36.3 years (standard deviation [SD] = 9.8). The numbers of participants who were married (50.1%) and single (including divorced and widowed; 49.5%) were almost equal. The average current work tenure was 11.0 years (SD = 9.1). More than half of the participants practiced in primary hospitals (58.2%), followed by secondary (24.6%) and tertiary hospitals (17.2%), and 78.0% of them came from private hospitals. As per the questionnaire survey, 19.4% of the participants worked in the emergency room; 15.5% worked in the medical ward; and 3.8% worked in other units, such as affiliated nursing homes, hemodialysis center, or oncology centers. The majority of our participants were nurses (72.2%) and doctors (9.1%); others (1.7%) included dietitians and infection control practitioners. They worked for an average of 43.8 h per week, and 7.4% of them had worked for more than 50 h per week in the month preceding this study. Some participants (26.4%) reported that they had encountered SARS before, and 31.4% of the participants cared for patients with confirmed or suspected COVID‐19.

Of the 1209 participants, 23.7% (*n* = 286) thought that he/she should not care for patients with COVID‐19 and 11.4% (*n* = 138) looked for another job or were considering resigning because of the risk of contracting COVID‐19. Participants who replied “yes” to the above two statements were regarded as seriously considering leaving their job. Among the voluntary participants, 7.8% (*n* = 94) harbored intentions to leave their job.

To better evaluate and group major components of the perceptions of the health care workers regarding their workplace environment and the risks of COVID‐19, 26 items of the questionnaire were analyzed through principal component analysis, supported by the KMO score (0.85) and Bartlett scores (chi square statistic = 12274.65; degrees of freedom = 325; *p* = 0.000). The analysis resulted in five factors with eigenvalues greater than 1.0 (Table S2). Oblique rotation (promax) was implemented assuming that the dimensions are correlated (Carpenter, 2018). The outcomes showed that oblique rotation grouped the items into five factors. The cumulative variability was 71.48%, as explained by these factors. From the 26 items, seven items, for example, “I feel that the protective measures at work are generally effective,” were reverse scored and grouped to indicate organizational support; other seven items were used to indicate perceived risk, for example, “I am worried I might transmit COVID‐19 to people close to me”; four items were grouped to represent affected social relationships, for example, “people avoid my family members because of my job”; four reverse‐scored items were grouped into supportive administration/management, for example, “I feel appreciated by society”; and the last four items were used to denote increased workload and job stress, for example, “I have an increased workload.”

Because the number of items differed among the five factors, the total score of each factor was adjusted between 25 and 100, and the 80th percentile was used as the cut point. For example, the 80th percentile of organizational support, perceived risk, social relationships, supportive administration/management, and increased workload and job stress were 53.57, 82.14, 62.50, 68.75, and 75.00, respectively. Health care workers with more than or equal to the 80th percentile scores of these factors were viewed as having low organizational support, high perceived risk, more affected social relationships, poor supportive administration/management, and high increased workload and job stress.

Table S3 presents the factors that predicted the possibility of the health care workers leaving their job. In the univariate logistic regression model, health care workers who were 26–35 years old; had worked for 5–10 years; practiced in primary or secondary hospitals; worked for >50 h a week; those who had ever cared for patients with confirmed/suspected COVID‐19; and those with low organizational support, high perceived risk, more affected social relationships, poor supportive administration/management, or highly increased workload and job stress had a higher possibility of leaving their job. The significant demographic characteristics and work‐related factors were then included in the multiple logistic regression. However, the current work tenure of the health care workers, instead of age, was retained in the model for multicollinearity (Katz, 2011), Bayesian information criterion, and Akaike's information criterion. The mutually adjusted model revealed that health care workers who had worked for 5–10 years at the time of participation in the study; those who practiced in primary or secondary hospitals; those who worked for >50 h a week; and those with a high perceived risk for contracting COVID‐19, more affected social relationships, poor supportive administration/management, or high increased workload and job stress had greater intentions to leave their job. The *R* ^2^ and area under the receiver operating characteristic curve of the adjusted model were 22.2% and 0.83, respectively.

## DISCUSSION

Compared with other countries (Dong et al., 2020), Taiwan has a lower fatality rate of COVID‐19 and most confirmed patients are migrants. During our survey from July to November in 2020, the confirmed cases increased from 448 to 675, and seven deaths remained unchanged (Taiwan Centers for Disease Control, 2021). This may affect the perceived risk of infection or death threat in Taiwan.

However, by April 17, 2021, health care workers had contributed to 14.29% of the 77 indigenous confirmed cases of COVID‐19 infection in Taiwan, which included two attendants, one cleaner, six nurses, and two doctors. This situation was quite similar to the situation at the end of the SARS epidemic, a 19.65% infection rate was observed among Taiwanese health care workers (Chan‐Yeung, 2004).

Under the circumstances of increased working hours, higher levels of exposure to illness, lack of adequate personal protective equipment, fear of getting infected with COVID‐19, and fear of transmitting the virus to others through close contact may affect the stress perception and mental health of health care workers (Kafle et al., 2021; Khajuria et al., 2021), which might be associated with increased intentions among them to leave their job (Norkiene et al., 2021). As the COVID‐19 pandemic progresses, provision of safe and appropriate workplaces for health care workers is also essential to ensure safe patient care (Centers for Disease Control and Prevention, 2021). Shiao et al. (2007) reported that approximately 7.6% of hospital nurses had seriously considered leaving their job during the SARS outbreak. The risk factors had included shorter job tenure, increased work stress, perceived risk of fatality from SARS, and affected social relationships. Kim (2018) reported that when the Middle East respiratory syndrome‐coronavirus (MERS‐CoV) rapidly spread in South Korea, nurses considered resignation because of the hospital system or because of the fact that it was mandatory for them to care for patients with MERS‐CoV. A total of 7.8% health care workers in our study had a serious intention to leave their job; this finding was similar to the observation of Shiao et al. (2007) during the SARS outbreak.

The original questionnaire was developed during the SARS episode in Taiwan by Shiao et al. (2007), and authorized to use in the present study. In this study in 2020, the content validity index and the test–retest reliability were examined, which turned out to be 0.97 and 0.64, respectively, indicating that the questionnaire was an acceptable research instrument. Principal component analysis was also used to examine construct validity for the 26 items in the questionnaire about the perception of their workplace environment and the risks of contracting COVID‐19. The participants' answers to these 26 items were grouped into five factors, namely *organizational support, perceived risk*, *affected social relationship*, *supportive administration/management*, and *increased workload and job stress*, which led to the cumulative variability of 71.48%. The Cronbach's *α*, a common measure of internal reliability, of the above five factors were 0.87, 0.82, 0.81, 0.80, and 0.75, respectively. Therefore, both the validity and reliability showed the 26‐items questionnaire was suitable for the investigation.

*Organizational support* comprises concepts of effective protective measures, clear policies and protocols, difficulty in adherence, resources for emotional support or personal protective equipment instructor, and confidence in employers' attendance polices if any of the health care workers contracted COVID‐19. The Taiwan government has adopted the principles of prudent action, rapid response, early deployment, and transparency to contain the spread of COVID‐19 (Chen, 2020). The Central Epidemic Command Center (CECC) was established to integrate resources, and it has held daily press briefings since January 2020 (Taiwan Centers for Disease Control, 2020). The CECC conveys information in a transparent manner and ordinates policies for community and health care facilities and distribution of personal protective equipment. It is important to have transparent and fair rules for abating the impacts of the pandemic (Braillon, 2021). *Perceived risk* involves the concept of the risk of contracting COVID‐19 and transmitting it to close contacts owing to the nature of the health care workers' job. Research focusing on health care workers has shown that health care workers often worry about getting infected themselves or transmitting the infection to their family (Awano et al., 2020; Kafle et al., 2021; Norkiene et al., 2021). *Affected social relationship* covers the concepts of undisclosed occupation and job contents and avoidance from others because of the job. Although Taiwanese individuals generally follow guidance and rules from the trustworthy government and maintain stability in the society, sporadic discrimination events were still reported by some health care workers and media. Incidences of discrimination and prejudice against health care workers and their families may come from incomplete information and fear toward the disease (Kim, 2018), consequently urging health care workers to consider changing their career (Shiao et al., 2007). *Supportive administration/management* is related to appreciation from the hospital and society, satisfactory staffing, and morale at work. Support from peers, organizations, and the society may mitigate the stress experienced during COVID‐19 (American Medical Association, 2021). *Increased workload and job stress* includes situations of overtime, increased workload, more stress, and more conflict among colleagues; these factors were found to be associated with a stronger ideation to leave among health care workers (Norkiene et al., 2021; Shiao et al., 2007).

By using 80th percentile as the cut point of each factor and after adjustment for demographic and work‐related factors, four out of five factors, namely high perceived risk, more affected social relationships, poor supportive administration/management, and increased workload and job stress, remained positively associated with the intention of health care workers to leave their job. In addition to the weekly working hours and work tenure, practicing in primary or secondary hospitals instead of tertiary hospitals was another predictor of health care workers leaving their job. This might be attributable to more resources and a better system established in tertiary hospitals.

To assess the robustness of our findings, we conducted sensitivity analyses, such as stratification of the participants by occupation, and then obtained approximate results as in the presented models.

The study has some limitations. First, the study design was cross‐sectional, and the results showed only the risk factors associated with the intention of health care workers to leave their job. Second, although the effective response rate was 88.9%, response bias may still have existed. Some health care workers replied that they were too busy to participate in the study when we invited them to participate in the study; thus, those who were not worried about the investigated issue might have also refused to respond to the survey.

## CONCLUSION

The present study indicates that a high perceived risk of COVID‐19, more affected social relationships, poor supportive administration/management, and high increased workload and job stress were positively associated with a serious intention to resign among health care workers. Therefore, mitigation of these risk factors might reduce the consideration of health care workers to leave to some extent, thus preventing workforce shortage and promoting patient safety. Further, hospitals should take care of their patients as well as their employees. Especially during this global pandemic, a follow‐up of the mental health of health care workers (Buselli et al., 2020) and provision of support to strengthen their resilience (Crowe et al., 2021) must be practiced to promote and create a supportive practice environment.

Our findings suggest that the intention of health care workers to leave their job during the pandemic was related to potentially modifiable factors relating to the infection itself and work environment. Development of appropriate workplace strategies is important to help mitigate these factors and promote workforce stability.

## CLINICAL RESOURCES

1. Keep health workers safe to keep patients safe: WHO, https://www.who.int/news/item/17‐09‐2020‐keep‐health‐workers‐safe‐to‐keep‐patients‐safe‐who
2. Caring for our caregivers during COVID‐19, https://www.ama‐assn.org/delivering‐care/public‐health/caring‐our‐caregivers‐during‐covid‐19
3. Help and support for healthcare workers—coronavirus (COVID‐19), https://www.dhhs.vic.gov.au/help‐and‐support‐healthcare‐workers‐coronavirus‐covid‐19

## Supporting information


Tables S1‐S3
