# Supplementary material for: Hospital factors that predict intention of health care workers to leave their job during the COVID‐19 pandemic
Source: J Nurs Scholarsh. 2022 Feb 20:10.1111/jnu.12771. Online ahead of print. doi: 10.1111/jnu.12771 (PMC9115187; doi:10.1111/jnu.12771)
Supplement: Supplementary file 1 — Tables S1‐S3 [file JNU-9999-0-s001.docx]

# Table S1. Participant demographics and work-related information (N = 1,209).

| Variable | n (%) | Mean (SD) |  |
| --- | --- | --- | --- |
| Sex |  |  |  |
| Male | 214 (17.7) |  |  |
| Female | 992 (82.1) |  |  |
| Missing | 3 (0.2) |  |  |
| Age (years) |  | 36.3 (9.8) |  |
| ≤25 | 148 (12.2) |  |  |
| 26–35 | 469 (38.8) |  |  |
| ≥36 | 581 (48.1) |  |  |
| Missing | 11 (0.9) |  |  |
| Marital status |  |  |  |
| Single/divorced/widowed | 599 (49.5) |  |  |
| Married | 606 (50.1) |  |  |
| Missing | 4 (0.3) |  |  |
| Current work tenure (years) |  | 11.0 (9.1) |  |
| <5 | 361 (29.9) |  |  |
| 5–10 | 326 (27.0) |  |  |
| >10 | 515 (42.6) |  |  |
| Missing | 7 (0.6) |  |  |
| Hospital level |  |  |  |
| Primary | 704 (58.2) |  |  |
| Secondary | 297 (24.6) |  |  |
| Tertiary | 208 (17.2) |  |  |
| Ownership of the hospital |  |  |  |
| Public | 266 (22.0) |  |  |
| Private | 943 (78.0) |  |  |
| Unit |  |  |  |
| Medical ward | 187 (15.5) |  |  |
| Surgical ward | 112 (9.3) |  |  |
| General ward | 54 (4.5) |  |  |
| Emergency room | 235 (19.4) |  |  |
| Intensive care unit | 162 (13.4) |  |  |
| Obstetrics, Pediatrics | 61 (5.0) |  |  |
| Community medicine/Nursing | 27 (2.2) |  |  |
| Radiology | 54 (4.5) |  |  |
| Laboratory | 34 (2.8) |  |  |
| Isolation ward | 8 (0.7) |  |  |
| Respiratory care ward | 22 (1.8) |  |  |
| Outpatient | 44 (3.6) |  |  |
| Rehabilitation | 32 (2.6) |  |  |
| Psychiatry | 81 (6.7) |  |  |
| Pharmacy | 41 (3.4) |  |  |
| Others | 46 (3.8) |  |  |
| Missing | 9 (0.7) |  |  |
| Occupation |  |  |  |
| Doctor | 109 (9.1) |  |  |
| Nurse | 873 (72.2) |  |  |
| Pharmacist | 60 (5.0) |  |  |
| Physical therapist | 29 (2.4) |  |  |
| Occupational therapist | 13 (1.1) |  |  |
| Respiratory therapist | 8 (0.7) |  |  |
| Medical technologist | 35 (2.9) |  |  |
| Radiologist | 42 (3.5) |  |  |
| Social worker | 20 (1.7) |  |  |
| Others | 20 (1.7) |  |  |
| Working hours/week |  | 43.8 (7.5) |  |
| ≤50 | 1,119 (92.6) |  |  |
| >50 | 90 (7.4) |  |  |
| Encountered SARS during this job |  |  |  |
| No | 888 (73.4) |  |  |
| Yes | 319 (26.4) |  |  |
| Missing | 2 (0.2) |  |  |
| Ever cared for patients with confirmed/suspected COVID-19 |  |  |  |
| No | 809 (66.9) |  |  |
| Yes | 380 (31.4) |  |  |
| Missing | 20 (1.7) |  |  |

SARS: severe acute respiratory syndrome

# Table S2. Factor loading of reported perceptions related to COVID-19.

|  | Factor | | | | |
| --- | --- | --- | --- | --- | --- |
|  | Organizational support | Perceived risk | Affected social relationship | Supportive administration/management | Increased workload and job stress |
| Eigenvalue | 5.88 | 3.90 | 2.35 | 1.73 | 1.56 |
| Variance explained (%) | 18.57 | 15.11 | 13.11 | 13.64 | 11.04 |
| Cumulative percentage | 18.57 | 33.69 | 46.80 | 60.44 | 71.48 |
| I feel that the protective measures at work are generally effective. | **0.84** | −0.02 | 0.03 | −0.08 | 0.03 |
| Clear policies and protocols were instituted for everyone to follow. | **0.82** | −0.02 | 0.02 | −0.03 | 0.04 |
| The policies and protocols were implemented quickly enough. | **0.80** | 0.02 | −0.03 | 0.10 | 0.04 |
| I had little difficulty in adhering to the recommended measures. | **0.80** | −0.05 | −0.04 | −0.03 | 0.09 |
| I have someone to turn to when I have a problem in using personal protective equipment. | **0.79** | 0.01 | 0.04 | −0.03 | −0.12 |
| Emotional support (e.g., counselling) is available to those who need help. | **0.75** | 0.09 | −0.07 | 0.14 | −0.05 |
| I am confident my employer will look after my medical needs if I were to fall ill with COVID-19. | **0.40** | −0.03 | 0.13 | 0.16 | −0.06 |
| I am worried I might transmit COVID-19 to people close to me. | −0.01 | **0.85** | −0.04 | 0.01 | -0.02 |
| I believe people close to me are at high risk of getting COVID-19 because of my job. | 0.01 | **0.83** | −0.01 | 0.00 | 0.02 |
| People close to me are worried for my health. | −0.04 | **0.79** | −0.15 | 0.04 | −0.04 |
| People close to me are worried they might get infected through me. | 0.02 | **0.76** | 0.19 | −0.08 | −0.07 |
| My family believes that I have a high risk of getting COVID-19. | 0.01 | **0.61** | 0.14 | −0.04 | 0.05 |
| I feel that my job puts me at great risk of exposure to COVID-19. | −0.06 | **0.58** | −0.05 | 0.05 | 0.16 |
| If I were to get COVID-19 I believe that my chances of survival are poor. | 0.14 | **0.32** | 0.24 | 0.00 | −0.02 |
| People avoid my family members because of my job. | −0.01 | 0.02 | **0.84** | −0.01 | −0.01 |
| People avoid me because of my job. | −0.07 | 0.05 | **0.80** | 0.09 | 0.01 |
| I avoid telling other people about the nature of my job nowadays. | 0.04 | 0.03 | **0.74** | 0.04 | 0.01 |
| I have been afraid of telling my family about the risk I am exposed to. | 0.08 | −0.02 | **0.74** | −0.11 | 0.02 |
| I feel appreciated by society. | −0.04 | −0.02 | 0.13 | **0.85** | −0.09 |
| The morale at work has been good. | 0.03 | 0.00 | 0.01 | **0.81** | 0.03 |
| I feel appreciated by the hospital/clinic/my employer. | 0.05 | 0.00 | −0.05 | **0.79** | 0.01 |
| There are adequate staff at my workplace to handle the different demands. | 0.10 | 0.02 | −0.08 | **0.67** | 0.07 |
| I have an increased workload. | 0.00 | 0.01 | −0.07 | 0.04 | **0.87** |
| I feel more stressed at work. | −0.04 | 0.10 | 0.01 | 0.01 | **0.78** |
| I have to work overtime. | 0.07 | 0.01 | 0.04 | −0.08 | **0.76** |
| There is more conflict among colleagues at work. | −0.05 | −0.11 | 0.37 | 0.05 | **0.46** |

# Table S3. Factors predictive of health care workers’ consideration of leaving their job.

|  | Univariate logistic regression | | | Multiple logistic regression | | |
| --- | --- | --- | --- | --- | --- | --- |
| Variable | Crude  OR | 95% CI | *P* value | Adjusted OR | 95% CI | *P* value |
| Sex |  |  |  |  |  |  |
| Male | 1 |  |  |  |  |  |
| Female | 0.90 | 0.54–1.59 |  |  |  |  |
| Age (years) |  |  |  |  |  |  |
| ≤25 | 1 |  |  |  |  |  |
| 26–35 | 3.19 | 1.36–9.32 | * |  |  |  |
| ≥36 | 2.17 | 0.92–6.38 |  |  |  |  |
| Current work tenure (years) | | | | | | |
| <5 | 1 |  |  | 1 |  |  |
| 5–10 | 2.10 | 1.18–3.82 | * | 2.10 | 1.08–4.22 | * |
| >10 | 1.52 | 0.87–2.72 |  | 1.03 | 0.53–2.05 |  |
| Marital status |  |  |  |  |  |  |
| Single/divorced/widowed | 1 |  |  |  |  |  |
| Married | 1.13 | 0.74–1.73 |  |  |  |  |
| Hospital level |  |  |  |  |  |  |
| Tertiary | 1 |  |  | 1 |  |  |
| Secondary | 2.52 | 1.12–6.45 | * | 4.49 | 1.74–13.37 | ** |
| Primary | 2.82 | 1.36–6.86 | ** | 5.51 | 2.21–16.12 | *** |
| Ownership of hospital |  |  |  |  |  |  |
| Public | 1 |  |  |  |  |  |
| Private | 1.41 | 0.83–2.54 |  |  |  |  |
| Occupation |  |  |  |  |  |  |
| Other health care workers | 1 |  |  |  |  |  |
| Nurse | 1.37 | 0.84–2.31 |  |  |  |  |
| Weekly working hours |  |  |  |  |  |  |
| ≤50 | 1 |  |  | 1 |  |  |
| >50 | 3.15 | 1.72–5.50 | ** | 2.59 | 1.21–5.30 | * |
| Encountered SARS during this job | | | | | | |
| No | 1 |  |  |  |  |  |
| Yes | 0.64 | 0.37–1.06 |  |  |  |  |
| Ever cared for patients with confirmed/suspected COVID-19 | | | | | | |
| No | 1 |  |  | 1 |  |  |
| Yes | 1.66 | 1.07–2.56 | * | 1.18 | 0.66–2.09 |  |
| Organizational support |  |  |  |  |  |  |
| High | 1 |  |  | 1 |  |  |
| Low | 1.95 | 1.23–3.04 | ** | 1.02 | 0.56–1.82 |  |
| Perceived risk |  |  |  |  |  |  |
| Low | 1 |  |  | 1 |  |  |
| High | 5.62 | 3.63–8.75 | *** | 3.58 | 2.14–6.00 | *** |
| Affected social relationships |  |  |  |  |  |  |
| Less | 1 |  |  | 1 |  |  |
| More | 4.48 | 2.92–6.92 | *** | 2.37 | 1.42–3.94 | * |
| Supportive administration/management | | | | | | |
| Yes | 1 |  |  | 1 |  |  |
| No | 2.93 | 1.90–4.51 | *** | 2.32 | 1.32–4.03 | *** |
| Increased workload and job stress | | | | | | |
| Low | 1 |  |  | 1 |  |  |
| High | 5.03 | 3.26–7.80 | *** | 2.87 | 1.68–4.87 | *** |
| SARS: severe acute respiratory syndrome  **P* < .05, ***P* < .01, ****P* < .001; OR, odds ratio; CI, confidence interval | | | | | | |
